# Effects of neighbourhood characteristics on the distribution of vacant houses in Toda city, a satellite city of Tokyo metropolis of japan

**DOI:** 10.1038/s41598-024-74663-3

**Published:** 2024-10-28

**Authors:** Qiyuan Liu, Kojiro Sho, Jonathan Corcoran, Naomi Ando

**Affiliations:** 1 Graduate School of Human-Environment Studies, Kyushu University, 744 Motooka, Nishi-ku, Fukuoka, Japan; 2 Department of Urban Engineering, School of Engineering, The University of Tokyo, 7-3-1 Hongo, Bunkyo-ku, Tokyo, Japan; 3 School of the Environment, Faculty of Science, The University of Queensland, Brisbane, QLD 4072 Australia; 4 Department of Architecture, Faculty of Engineering and Design, Hosei University, 2-17-1 Fujimi, Chiyoda-ku, Tokyo, Japan

**Keywords:** Vacant house, Neighbourhood characteristics, Satellite city, Residence promotion district, Multiscale geographically weighted regression, Environmental sciences, Environmental social sciences

## Abstract

As Japan faces a growing crisis of vacant housing, this study investigates the underexplored relationship between vacant house distribution and neighbourhood characteristics in Toda City, a satellite city of the Tokyo metropolis located in Saitama Prefecture. We selected 10 community features, including living convenience, housing supply, and policy support, to explore their influence on vacancy rates. Using a comprehensive dataset primarily derived from the PLATEAU data, we employed a two-step method combining global and local Moran’s I analyses to assess the spatial distribution normality of vacant houses. We then compared traditional statistical data analysis with spatial data analysis through multiscale geographically weighted regression to evaluate their effectiveness and identify the factors most closely associated with vacancy rates. Our findings reveal that spatial data analysis provides superior insights compared to traditional statistical methods. Both approaches consistently indicate a significant positive correlation between the supermarket area ratio and vacancy rates, and a significant negative correlation between road adjacency and vacancy rates. This study demonstrates the feasibility of using spatial data to analyse neighbourhood characteristics in the context of declining vacancy rates in satellite cities. The insights gained offer theoretical support for selecting data and methodologies in future research and policymaking that are aimed at mitigating the growth of vacant houses.

**Supplementary Information:**

The online version contains supplementary material available at 10.1038/s41598-024-74663-3.

## Introduction

The growing prevalence of vacant houses, driven by societal challenges such as declining birth rates, aging populations, and surplus housing stock, has become a critical issue for urban development in Japan and other countries (Gu & Asami, 2016; Suzuki & Hino, 2018)^1,2^. These vacant properties generate negative externalities, affecting not only the surrounding environment but also the character of residential neighbourhoods and local land values (Benediktsson, 2014; Sadayuki et al., 2020)^3,4^. In light of contemporary social shifts, the need to redistribute populations and resources between central and satellite cities has gained increasing attention (Westerink et al., 2013; Lee et al., 2015; Mouratidis, 2019)^5–7^. He and Zhang (2023)^8^, examining residential mobility in Beijing, China, found that because homes in satellite cities are often larger than those in central areas, residents of satellite cities are more likely to stay in their homes rather than relocate to urban centres. Similar trends have been observed globally, including in France (Segú, 2020)^9^, the United States (Myers et al., 2023)^10^, and South Korea (Park et al., 2021)^11^.

Compared to the complex urban dynamics of central cities, the vacant house phenomena is more widespread and easily identifiable in satellite cities (Hino et al., 2022; Nishiyama, 2020)^12,13^. However, most recent research on vacant houses has primarily focused on central cities within global metropolitan areas, such as metropolitan counties in the United States (Zhu et al., 2024)^14^; Shenzhen (Liang, 2021)^15^, Guiyang (Shi et al., 2022)^16^, Chengdu (Zhang et al., 2023)^17^, and Shenyang (Williams et al., 2019)^18^ in China, as well as Tokyo (e.g., Sadayuki et al., 2020)^4^ and Utsunomiya city (Nishiyama, 2020)^13^ in Japan. While some studies (e.g., Baba & Asami, 2017; Baba & Hino, 2019)^19,20^ have addressed the issue of vacant houses in satellite cities, there remains a significant gap in the empirical analysis of vacant house distribution in relation to neighbourhood characteristics. Understanding the relationship between neighbourhood characteristics and vacant houses is crucial for identifying the factors that influence people’s decisions to live in satellite cities. Key factors affecting residential satisfaction include the availability of green spaces, accessibility to public transportation, and proximity to urban public spaces. Overall living convenience is also a critical consideration (Tao et al., 2022; Shen et al., 2021; Chiang et al., 2015; Zhu & Wang, 2023; Kikuchi et al., 2022)^21–25^, and these preferences can vary significantly across age groups (Zhuang & Ye, 2023)^26^. Scholars (e.g., Szabó et al., 2014; Ouyang, 2017)^27,28^ have highlighted that residents are less likely to choose less convenient areas for their homes, suggesting that living convenience may play a key role in the occurrence of vacant houses in satellite cities.

Vacant housing arises from a mismatch between housing supply and demand (Molloy, 2016; Madanipour, 2018)^29,30^, highlighting the important role played by housing supply in the emergence of vacant homes. In Japan’s satellite cities, housing supply issues such as residents relocating due to inadequate living space or the inability to find tenants have already been noted, which indicates the considerable impact of housing supply on the occurrence of vacant homes. In satellite cities in Japan, housing supply issues such as residents moving owing to insufficient living space or an inability to find tenants, have been observe (Toda City, 2018, 2023)^31,32^. Studies from East Asian countries further indicate that unit floor area is a key factor contributing to the rise in vacant houses, particularly in China (Yang et al., 2023)^33^, Japan (Baba & Hino, 2019)^20^, and Taiwan (Chiang et al., 2015)^21^. However, the specific floor area most associated with vacancy rates has yet to be clearly identified. 

Policy also plays a significant role in the prevalence of vacant houses. For instance, Cho et al. (2020)^34^ found that the implementation of urban renewal plans had a considerable impact on residential property values, which may lead to a reduction in vacant homes in surrounding areas. In Mexico, Monkkonen (2019)^35^ revealed that federal housing finance policies were linked to higher vacancy rates, a trend observed in both central and satellite cities. Similar policy interventions have been explored in South Korea (Park et al., 2021)^11^ and Taiwan (Hsiao, 2022)^36^, suggesting that policy support may be a crucial factor influencing vacancy rates in satellite cities. 

This review suggests a lack of comprehensive understanding regarding the relationship between vacant house distribution and neighbourhood characteristics, such as living convenience, housing supply, and policy support, in satellite cities. By clarifying how these factors impact vacant house occurrences, this study seeks to uncover the underlying mechanisms driving the phenomenon of vacant homes in satellite cities.

## Literature review

### Vacant housing scholarship

Within the broad global discussions on vacant housing, there are various aspects of interest, including the methods used for identifying vacant properties. In China, Zou and Wang (2020)^37^proposed an algorithm to distinguish between vacant and occupied residential properties using remote sensing image processing and analysis techniques. Similarly, Williams et al. (2019)^18^ developed a method based on social media data method to identify the distribution of vacant houses in China. These discussions also encompass interpretations of the status of vacant properties, such as those using spatial autocorrelation indicators. For example, Morckel (2014)^38^ identified statistically significant clustering of housing vacancies and predictors of abandonment in the U.S. Further, Molloy (2016)^30^ provided valuable insights into vacant houses in the U.S. real estate market.

In this study, we focused on the occurrence and distribution of vacant housing across different countries to bolster the theoretical framework of our research. Previous comprehensive nationwide analyses have addressed the distribution of vacant houses and strategies for reducing vacancy rates in various countries, including South Korea (e.g., Joo et al., 2022; Jeon & Kim, 2023)^39,40^, Taiwan (e.g., Hsiao, 2022)^36^, Syria (e.g., Wind & Ibrahim, 2020)^41^, and France (e.g., Segú, 2020)^9^. Our extensive literature review indicates that discussions about the distribution of vacant housing are not uncommon worldwide. The following section focuses on exploring the literature on vacant housing within the Japanese context.

### Vacant housing scholarship in Japan

According to the Ministry of Land, Infrastructure, Transport and Tourism (MLIT, 2015)^42^, the Japanese government defines vacant housing as buildings or related structures that are not used for residential purposes. According to data published by the Statistics Bureau, Ministry of Internal Affairs and Communications (2019)^43^, the number of vacant houses in Japan increased from 57.59 million in 2008 to 62.42 million in 2018, with the vacancy rate rising to 13.6%. The Special Measures Act on the Promotion of Vacant House Countermeasures was enacted in 2014 to curb this increasing trend.

Cutting-edge research on the identification of vacant housing in Japan has been advancing towards more predictable and convenient methods. For example, Tomita et al. (2022)^44^ employed machine learning techniques for analysis, and Konomi et al. (2019)^45^, used Wi-Fi network usage data. This shift towards innovative approaches is a response to the mainstream method for investigating vacant houses in Japan, which primarily relies on information on registered vacant houses and water meter usage data (e.g., Akiyama et al., 2018; Sayuda et al., 2022)^46,47^. This limits the understanding of the relationship between the distribution of vacant houses and neighbourhood characteristics (Ouchi, 2018)^48^.

In Japan, the research on vacant houses is concentrated in large cities. The excessive dependence on and concentration of population and wealth in Tokyo city centre have been mitigated by surrounding satellite cities in Chiba, Saitama, and Kanagawa Prefectures (Kidokoro et al., 2023; Gu et al., 2023)^49,50^,. Nevertheless, the number and scale of vacant houses are highest in large cities in Japan (Statistics Bureau, 2019)^43^, meaning that most studies have focused on cities such as Tokyo (e.g., Gu & Asami, 2016; Sadayuki et al., 2020)^1,4^. This scenario highlights a clear research gap and indicates the necessity of studying vacant houses in satellite cities. There is an awareness of the significance of vacant housing in Japan and the related research gap. Consequently, previous studies have conducted preliminary assessments of vacant houses in Japan using big data and public data and have identified indicators related to neighbourhood characteristics.

### Relationships between urban neighbourhood characteristics and vacant houses

The correlation between neighbourhood characteristics and vacant houses has been rarely studied, although several scholars (e.g., Hackman, 2019; Chen, 2023)^51,52^ have discussed the importance of neighbourhood characteristics for quality of life. Yamada et al. (2016)^53^ revealed that residents prioritise the convenience of shopping, public transportation, commuting, scenic street views, and environmental hygiene as essential neighbourhood characteristics. Moreover, using ordinary least squares and logistic regression models, Deng and Ma (2015)^54^ demonstrated a correlation between vacant houses and local amenities such as supermarkets, retail stores, bus stations, and parks. Basic infrastructure, public facilities, and parks have also been found to be associated with residents’ housing awareness and occupancy rates (Gao & Asami, 2007; Liu et al., 2015; Shi et al., 2023)^55–57^.

As shown in Table 1, among the various factors influencing the occurrence of vacant houses, studies have emphasised the importance of building size, road conditions, and facilities within the urban structure, demonstrating their positive or negative relationships with the distribution of vacant houses (Oda et al., 2018; Baba & Asami, 2017; Baba & Hino, 2019; Joo et al., 2022; Williams et al., 2019; Gu et al., 2019; Kim et al., 2016)^18–20,40,58–60^. Based on a systematic literature review, this study identified the factors that are potentially associated with the occurrence of vacant houses, including living convenience and housing supply.

**Table 1:** Relationship between neighbourhood characteristics and vacant house distribution.

Author	Study Area	Method	Verified variables
Oda et al. (2018)	Hadano, Japan	Structural equation model	+Distance to educational facilities
Baba and Asami (2017)	Three major metropolitan areas in Japan	Logistic regression model	+Floor area +Adjacent to a road +Grocery stores ­Daycare facilities
Baba and Hino (2019)	Kawaguchi, Japan	Multinomial logistic regression	+Adjacent to a road ­Floor area
Joo et al. (2022)	South Korea	Logistic regression model	­Contact area (adjacency)
Williams et al. (2019)	China	Cluster analysis	+Distance to school
Gu et al. (2019)	Dayton, U.S.	Regression analysis	­Retail lots (facilities) +Floor area
Kim et al. (2016)	Nagasaki, Japan	Logistic regression	+Distance from educational facilities

*+&­indicate a positive and negative correlation with the distribution of vacant houses, respectively.

## Research framework

### Research purpose and study area

**Fig. 1:**
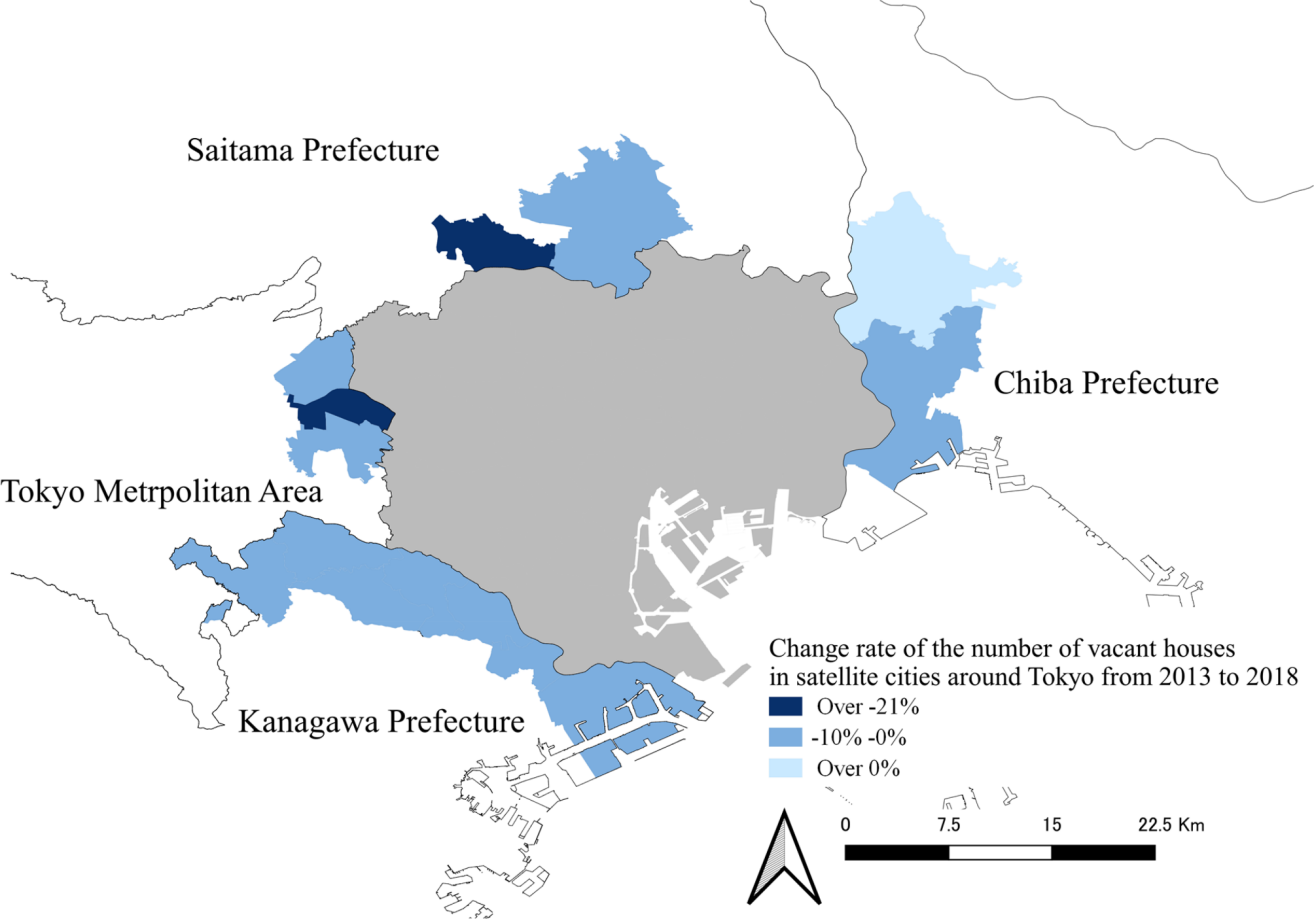
Changes in the number of vacant houses in satellite cities of Tokyo metropolis from 2013 to 2018 (Source: Created by the authors using QGIS 3.34.10).

Despite an overall upward trend in Japan’s house vacancy rate, there are distinct regional and municipal variations characterising this phenomenon, and certain areas with different changes in vacancy rate drew our attention. According to Statistics Bureau (2019)^43^, regional cities such as Wakayama Prefecture observed a 2.3% increase in their vacancy rate from 2013 to 2018. In contrast, the vacancy rate in Saitama Prefecture, a suburb of Tokyo, decreased from 9.1 to 8.4% over the same period (Saitama Prefecture Government, 2023)^61^. Satellite cities also displayed a trend of decreasing house vacancies in comparison to other metropolitan regions. As shown in Fig. 1, most satellite cities near Tokyo experienced a decreasing trend in the number of vacant houses from 2013 to 2018. For instance, in Toda City, there was a relative decrease of -20.67% in the number of vacant houses over five years. While Musashino City’s decreasing vacant houses are notable, its inclusion in the Tokyo metropolis likely mirrors the dynamics of larger urban areas.

Considering the unique characteristics of roads in Japanese cities, the distance between houses and roads is considered an influencing condition for the occurrence of vacant houses and the specifics of local policy support. Employing three analytical perspectives and 10 influencing factors, this study endeavoured to create a correlation model for the distribution of vacant houses in Toda City. The study innovatively examined the distribution of vacant houses using data from Japan’s MLIT.

In 2023, Toda City had a population of 1.42 million people, which is comparatively small compared to the top three cities in Saitama Prefecture: Saitama City (13.25 million people), Kawaguchi City (5.94 million people), and Kawagoe City (3.55 million people). Despite the overall decline in population, Toda City demonstrates an upward trend, as indicated in the Toda City Urban Transportation Master Plan (2019). According to data released by the Toda City government in 2023, the vacancy rate decreased from 10.9% in 2013 to 8.4% in 2018, ranking second lowest in Saitama Prefecture. Thus, compared to other regions, Toda City exhibits a distinctive dynamic regarding vacant housing.

**Fig. 2:**
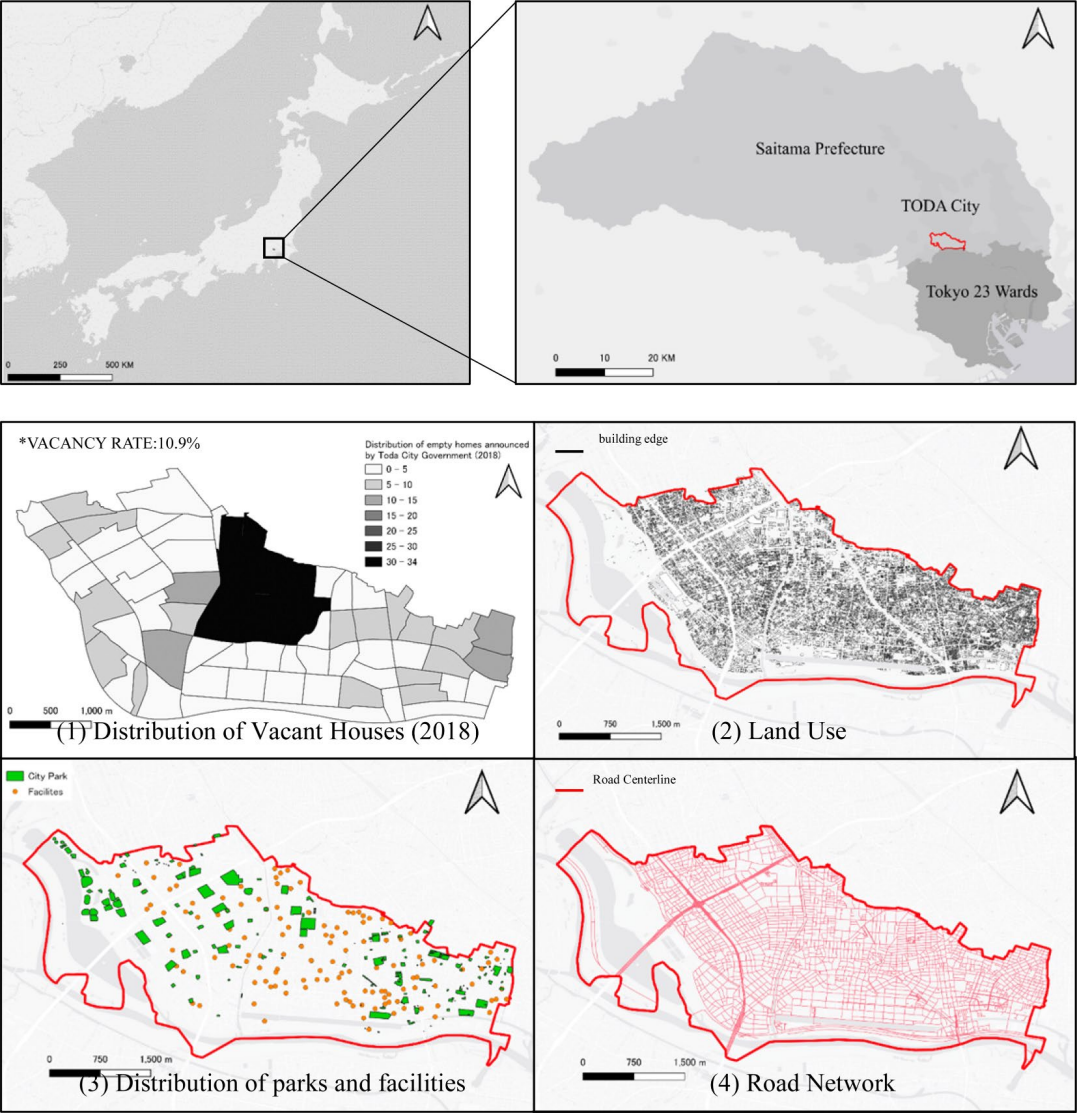
Characteristics of Toda City. (Source: Created by the authors using QGIS 3.34.10).

Considering this data, we selected Toda City, as a satellite city of Tokyo metropolis, our research setting to investigate the occurrence of vacant houses within an urban context in Japan. This choice was designed to facilitate a comprehensive understanding of the reasons for and mechanisms behind the changes in vacant houses. Figure 2 shows the distribution of vacant houses and major neighbourhood characteristics, including land use, the distribution of public facilities, and the road network, in Toda City. A survey by the Toda City Government in May 2018 identified 263 vacant houses in the city. Vacant houses were more highly concentrated in some areas, as shown in panel (1) of Fig. 2.

This study aimed to contribute to the knowledge on the increasing number of vacant properties both globally and across Japan by using traditional statistical data analysis and spatial data analysis for comprehensive comparison and cross-verification. We sought to determine which factors are significant and their status to clarify the influencing mechanism behind the decreasing trend of vacant houses in Toda City. We also compared the overall model results and spatial analysis to determine which method is more suitable for analysing vacant houses using urban data. Our results provide insights for future efforts to identify key factors and suitable methods for curbing vacant housing in Japan.

### Research methodology

We selected both statistical data analysis and spatial data analysis methods. In terms of statistical data analysis, we followed, Shi (2022)^16^ and Segú (2020)^9^, who underscored the feasibility of linear correlation and the significance of p-values generated through t-tests for analysing vacant house distribution. Additionally, considering the suboptimal model fit, we employed a statistical visualisation method, the discretisation scatter plot proposed by Cabitza and Campagner (2019)^62^, to examine the distribution intervals and concentration trends of the factors most correlated with vacant houses. Based on this, we conducted correlation analysis and multiple regression analysis of the various factors using the statistical data.

The spatial data analysis began with a spatial autocorrelation analysis including both global and local Moran’s I analysis. The feasibility of this method for studying vacant houses has been demonstrated by Williams et al. (2019)^18^ and Lee and Newman (2021)^63^. This approach helps elucidate the distribution patterns of vacant houses.

Following the preliminary examination of whether the distributions of vacant houses exhibited spatial clustering based on global and local Moran’s I analysis, we employed multiscale geographically weighted regression (MGWR) to empirically analyse its feasibility in urban data studies (Yin et al., 2022; Lu et al., 2023; Yu and Lee, 2023)^64–66^. We used MGWR to determine the impact of various factors on the distribution of vacant houses in different areas within Toda City and to evaluate the spatial model fit for each factor.

Following the methods proposed in the abovementioned research, the major novelty of our study is its proposal of a two-step method for examining vacant houses. In this two-step method, an initial correlation analysis examines the linear relationships between various urban factors and housing vacancies. Subsequently, a multiple regression analysis quantifies the impact of these variables. MGWR enhances this analysis by accounting for spatial heterogeneity, thereby improving the model’s accuracy in representing the neighbourhood characteristics of Toda City. The visualisation of the data on the most influential factors identified by MGWR provides deeper insights into their spatial distribution.

Overall, we employed two similar research methods, seeking to compare the results of statistical data analysis with those of spatial data analysis. This approach aimed to determine which model better explained the distribution of vacant houses in Toda City according to the selected neighbourhood characteristics. This comparative analysis framework enabled us to gain a deeper understanding of the mechanisms underlying patterns in vacant house distribution (e.g., Zhang et al., 2023; Zhu et al., 2024; Yılmaz, 2023)^14,17,67^.

### Neighbourhood characteristics associated with vacant houses

#### Living convenience variables

First, from the perspective of living convenience, this study mainly selected the facility building, service range of bus stations, and facility configuration of each town, a total of five influencing factors. The MLIT survey revealed resident concerns regarding commuting facilities, support for older adults, and child support. The survey selected five types of facilities that had been clarified as important in previous studies: convenience stores, large supermarkets (Jeon & Kim, 2020)^68^, kindergartens (Theisen, 2017)^69^, parks (Gai et al., 2023)^70^, and elderly support facilities (Kwon, 2017)^71^.

To delineate the service range for public facilities, we referred to research by NILIM (2021), which categorised daily convenience facilities into five types: commercial facilities selling fresh produce, stores for daily goods, older adult care facilities, childcare facilities, and office spaces. We determined a service range of 300 m for two primary reasons. First, research within Japan (e.g., Chosokabe & Ogawa, 2016)^72^ indicates that residents prefer to live within 300 m of public facilities. Similarly, international studies (e.g., Chiang et al., 2015)^21^ suggest that adjusting the service range of convenience stores better suits local contexts. The second reason is that a 500-meter service range is too large in the context of Toda City, where it would lead to an excessive number of residential building samples within that range, which might skew the statistical analysis results. Based on these considerations, residential properties located more than 300 m from any of the five public facilities were considered more likely to be vacant, with this influencing factor denoted as 300 F/N.

Determining that the service range of a bus station is 200 m, we considered the number of buildings within the service range of a bus station in each town of Toda City as an influencing variable of living convenience, denoted as B/N. Discussed as one of the *service range*, the scope of services is considered.

For a more in-depth analysis of the living convenience across various types in Toda City, we also employed the area ratio method as an independent variable. This variable was derived by dividing the area of supermarkets, convenience stores, and park facilities in each town by the area of houses in that town, represented as S/F, C/F, and P/F, respectively. Additionally, adhering to the definition in the Urban Structure Assessment Manual (MLIT, 2014)^42^, discussing the *service area* in terms of convenience.

#### Housing supply variables

**Fig. 3:**
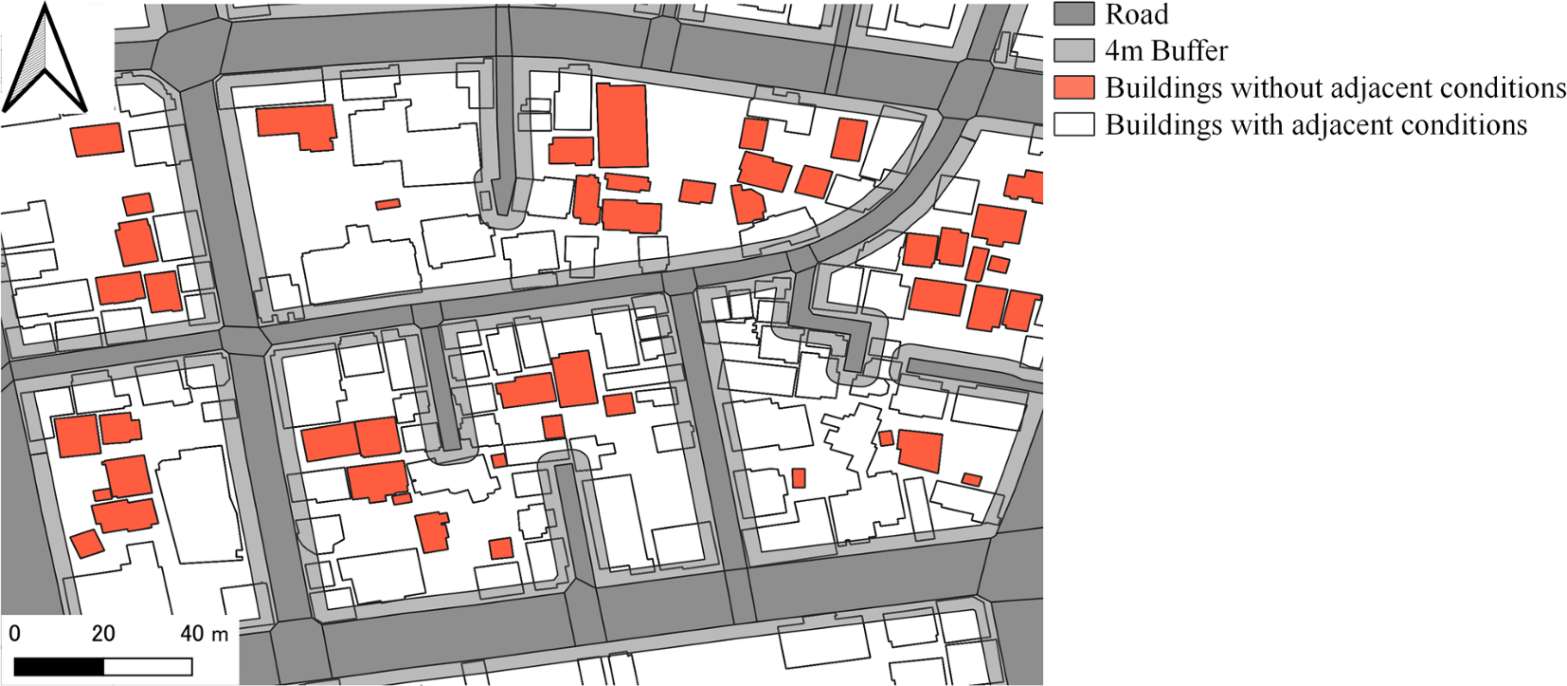
Criteria for determining adjacent conditions.

To analyse housing supply, this study primarily considered two influencing factors: floor area and access conditions. Concerning road adjacency, the greater the distance between a house and the road, the poorer the living and site conditions of the house, and the higher the likelihood of it being vacant. In this study, the criterion for determining a house’s access conditions was based on the premise that a building is road adjacent if either of its sides is close to a certified road. Buildings not connected to certified roads are classified as disconnected structures and are exempt from road proximity requirements. However, assessing a building’s road adjacency merely by visually examining publicly available map data is insufficient. As illustrated in Fig. 3, Usui and Asami (2010)^73^ proposed a GIS method for determining touchpoints using buffers, with the optimal buffer distance set at 4 m. We employed QGIS software to establish a 4-meter buffer zone starting from the road boundary. Following the logical premise of road adjacency, buildings intersecting with the road buffer zone were classified as connected, whereas those not intersecting were labelled as disconnected. The proportion of buildings with road access in each town in Toda City was designated as A/N, serving as one of the factors of *access conditions*.

For the dwelling’s floor area, the GIS data used determined that residential buildings with an area of 15 to 150 square meters should be included, focusing the analysis on residential structures. However, the geometric area represented in publicly available building data from PLATEAU reflects the area projected by the eaves. To enhance the accuracy of the identification of vacant houses in Toda City, this study categorised floor areas into three groups: 15–64 square meters, 65–99 square meters, and 100–150 square meters. The proportion of *floor area* in each town was calculated for these categories, establishing the impact factors as 15–64/N, 65–99/N, and 100–150/N.

#### Policy support

This study incorporated the current residence promotion district (RPD) area published by the MLIT as a judgment condition to consider policy support. When the Japanese government revised the Act on Special Measures Concerning Urban Renaissance in 2014 in the face of a shrinking population and urban constraints, specific areas were designated as part of the RPD to maintain a certain population density, as illustrated in the upper part of Appendix 1. The relationship between vacant house distribution and the RPD policy is crucial; therefore, whether each town was part of the RPD was considered an evaluation criterion in this study. According to the Toda City Land Adaptation Plan announced by the local government (bottom of Appendix 1), the total RPD area in Toda City covered 1.086 hectares in 2019, constituting 81% of the RCD. Considering that the residential inducement area is not demarcated by town boundaries, the area ratio in each town was calculated binarily. If the residential inducement area was below 50%, the ratio was 0. If it exceeds 50%, the ratio was 1.

This study’s research methodology involved analysing 10 parameters related to housing supply and living convenience as independent variables, with policy support acting as the control variable for the final analysis results and the distribution of vacant houses in Toda City.

### Research data

**Fig. 4:**
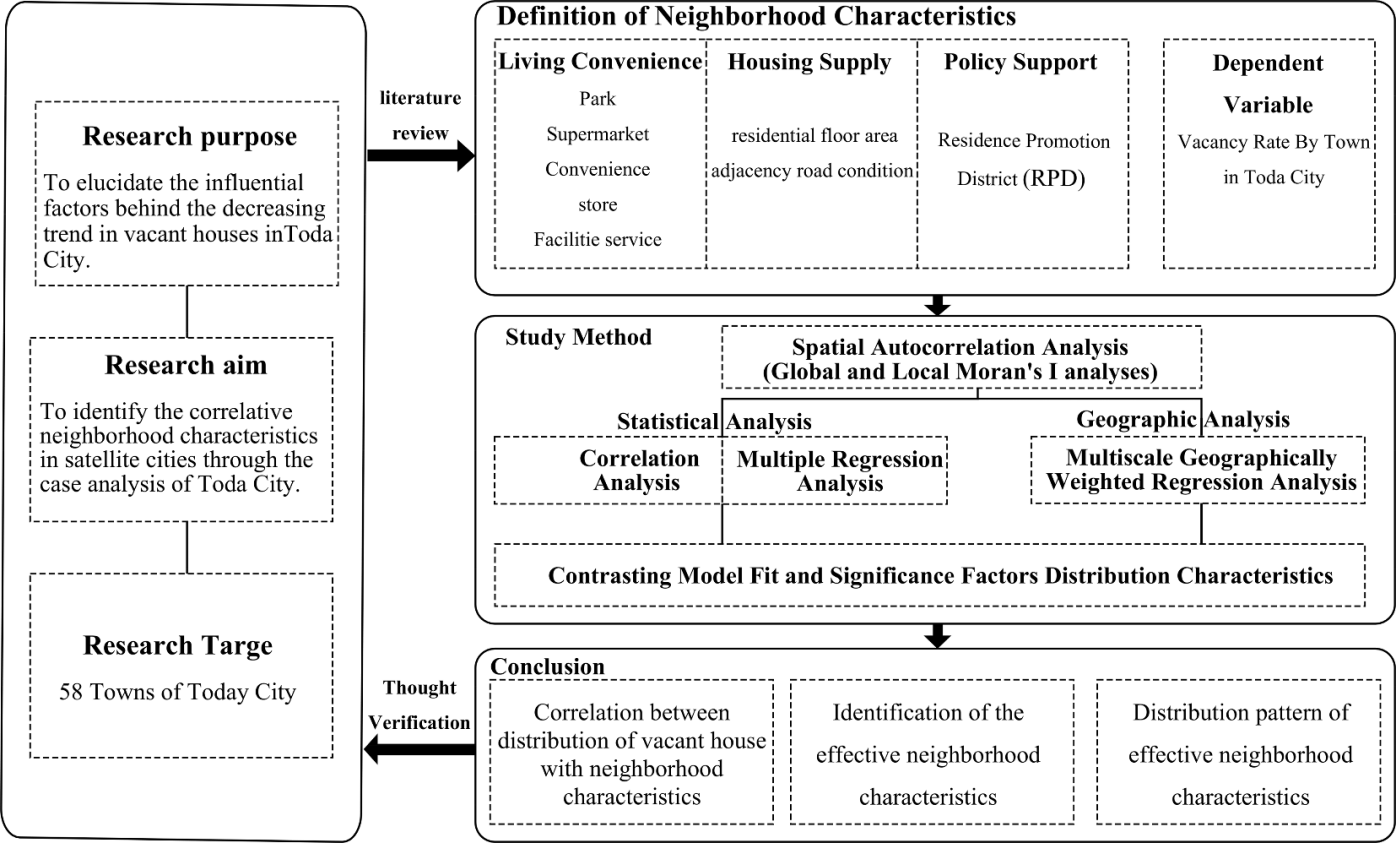
Research process and framework.

A thorough examination of the existing literature and case studies reveals numerous investigations into the correlation between vacant houses and neighbourhood characteristics such as transportation and facilities. Park et al. (2021)^11^, who compiled factors related to vacant houses in Korean cities, emphasised population density, ageing, urban structure, housing size, facility distribution, and alleyway layouts. Samoto and Miyagawa (2021)^74^ highlighted the impact of urban transportation networks and the development of public transportation facilities on vacant house distribution, focusing on factors like building density, age, and proximity to roads and schools. Ardeshiri (2018)^75^ underscored the significance of residents’ perspectives in their choices of urban facilities. Figure 4 presents this study’s research process and framework.

In selecting data, this study specifically chose indicators related to neighbourhood characteristics from urban indicators guiding functions and floor area (MLIT, 2014)^42^. Subsequently, factors associated with the distribution of known vacant houses in Toda City (Fig. 2) were extracted and a comprehensive analysis of their relationship was conducted and verified. Given that the published distribution of vacant houses is organised by the number of vacant houses in each town (Table 2), this study analysed the living convenience of each of the 58 towns in Toda City by reorganising 10 relevant variables into an impact variable model. The town level, referred to as ‘Chome’ in Japanese, serves as the smallest statistical unit for the census and most public statistic surveys (Kidokoro et al., 2023)^50^. Through this process, this study aimed to enhance the understanding of how policy support influences the relationship between vacant housing, living convenience, and housing supply and confirm their correlation with the distribution of vacant housing.

The data used in this study was drawn from the latest map data developed by the MLIT, named PLATEAU (https://www.mlit.go.jp/plateau/). PLATEAU was selected because, in comparison to other publicly available government geographic information, it offers more detailed information, such as building area, height, and land use, than other datasets. This choice significantly reduced the time required for data analysis and computation, facilitating the research process. From the 2022 edition of the PLATEAU data, this study directly selected geographical information on building area and facilities, the distribution of public facilities, the administrative boundaries of towns, and road information. As shown in Table 3, following the methods described in Sect. Neighbourhood characteristics associated with vacant houses for data sourcing and computation, we calculated the proportion of residential areas outside service areas, the proportion of houses with adjacent roads in each town, the ratio of facility areas to residential areas, and other factors, resulting in a total of 10 variables.

**Table 2:** Definition of neighbourhood characteristics and summary of data.

Neighbourhood characteristics	Data perspective	Influencing factor	Abbreviation	Type of Data
Living convenience	Service range	Houses outside a 300-meter service radius of all public facilities in each town	300 F/N^*^	Numerical ratio
	Service area	Ratio of the total residential floor area of a supermarket to residential floor area in each town	S/F^**^	Area ratio
		Ratio of the total residential floor area of the park area in each town	P/F	Area ratio
		Ratio of the total area of a convenience store to residential floor area in each town	C/F	Area ratio
		Proportion of houses within a 200-meter service radius of a bus stop in each town	B/N	Numerical ratio
Housing supply	Access conditions	Proportion of houses with adjacent roads in each town	A/N	Numerical ratio
	House area	Proportion of residential floor area in each town between 15 and 64 square meters	15–64/N	Numerical ratio
		Proportion of residential floor area in each town between 65 and 99 square meters	65–99/N	Numerical ratio
		Proportion of residential floor area in each town between 100 and 150 square meters	100–150/N	Numerical ratio
Policy support	Policy perspective	Residential-induced regional planning	RPD	Numerical ratio

* N is the sum of the number of houses in Toda City;.

** F is the sum of floor area in Toda City.

## Analysis results

Following the research methods and procedures outlined in Sect. 3.4, we first collected and processed the selected data. After obtaining the data characteristics, we conducted statistical data analysis and an analysis of the vacant house data, and then compared the two. We first analysed the data characteristics of the 10 variables of neighbourhood characteristics in Toda City, as shown in Table 3.

**Table 3:** Main characteristics of the variables of neighbourhood characteristics in Toda City(*N* = 58).

Analysis perspective	Influencing factors	Minimum	Maximum	Mean	Std. Deviation
	VH/N*	0.00	0.05	0.0095	0.00827
Living convenience	300 F/N	0.00	0.89	0.2233	0.24836
	S/F	0.00	0.45	0.0229	0.07224
	P/F	0.00	7.76	0.1982	1.01274
	C/F	0.00	0.01	0.0016	0.00282
	B/N	0.20	1.00	0.8664	0.17728
Housing supply	A/N	0.17	0.58	0.3331	0.07439
	15–64/N	0.03	0.68	0.3722	0.10168
	65–99/N	0.05	0.29	0.2059	0.05658
	100–150/N	0.05	0.20	0.1292	0.03504
Policy support	RPD	0	1	0.83	0.381

*VH/N: Vacancy rate in each town.

### Moran’s I results

To assess the distribution of vacant houses in Toda City, this study incorporated spatial autocorrelation analysis, examining whether the vacancies exhibited distinct spatial characteristics across various neighbourhoods. The study utilised both global and local Moran’s I analysis to dissect the distribution trends and their significance, with findings depi e of 3.981130 and a significantly low p-value of 0.000069 (Table 4). These results suggest that there is less than a 0.01% probability that this spatial pattern is the result of random chance, indicating that the spatial distribution of vacant houses in Toda City is not random but significantly clustered.

**Fig. 5:**
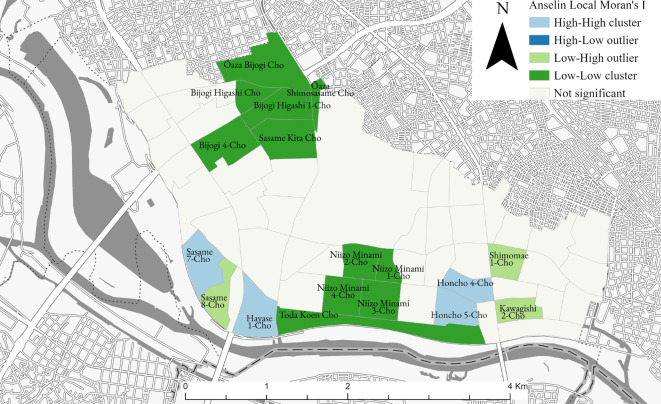
Local spatial autocorrelation distributions of vacant house rates in each town of Toda City.

The Local Moran’s I provided more nuanced insights into these patterns, indicating specific neighbourhoods where vacant houses are concentrated. Figure 5 illustrates areas of clustering where vacant houses were significantly more prevalent than the average (High-High cluster), such as Sasame 7-chome and Hayase 1-chome, with a total of four chomes located in the southern area of Toda City. The figure also shows clusters where vacant houses are less prevalent than the average (Low-Low cluster), such as Ōaza Bijogi cho and Bijogi Higashi 2-chome, which are clustered in the northwestern direction of Toda City. In addition, it highlights spatial outliers where a high prevalence of vacant houses is surrounded by areas with a low prevalence (High-Low outlier) and vice versa (Low-High outlier), for example, Sasame 8-chome.

Based on these results, we can conclude that certain areas in Toda City exhibit statistically significant clustering of vacant houses. The presence of statistically significant clustering in specific areas implies that random chance alone does not account for the spatial distribution of vacant houses, suggesting the influence of underlying neighbourhood facts.

**Table 4:** Summary of global Moran’s I results.

Moran’s Index	0.256172
Expected Index	-0.017544
Variance	0.004727
z-score	3.981130
p-value	0.000069

### Statistical data analysis results

#### Linear correlation between vacant houses and neighbourhood characteristics

This study treated the vacancy rate in each town as the dependent variable, examining other factors as independent variables. A 95% confidence interval was set for the statistical results. This confidence level was used to determine the significance of the correlations, highlighting the robustness of the results for factors where *p* < 0.05 and *p* < 0.01.

**Fig. 6:**
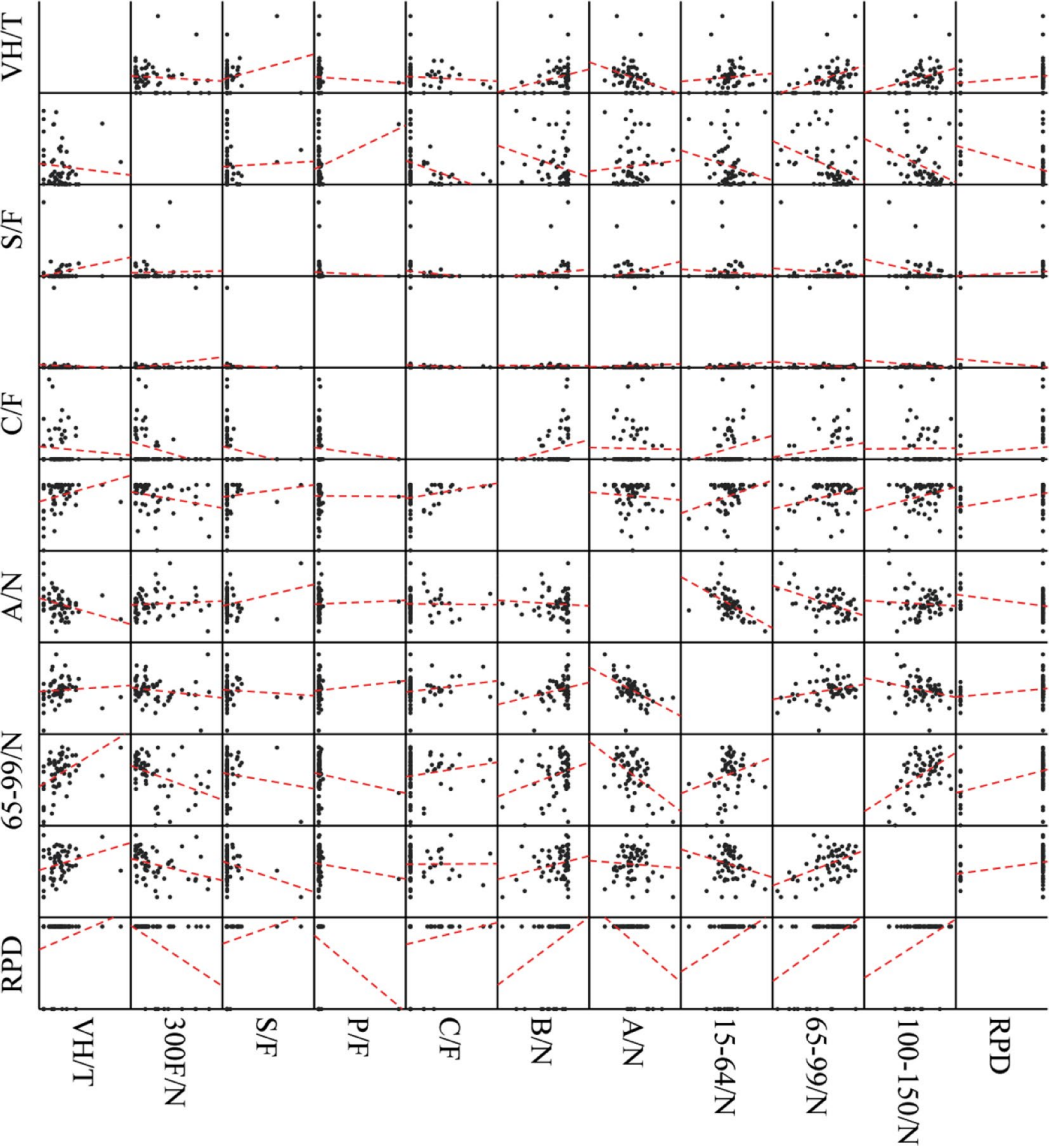
Linear correlation results for each variable of neighbourhood characteristics.

The correlations between the 10 independent variables and the vacancy rate in each town are presented in Table 5; Fig. 6. The variable 65–99/N exhibited the strongest correlation with the vacancy rate in each town, followed closely by the floor area of 100–150, which demonstrated a similar correlation. Other factors, such as 300 F/N, did not show a direct correlation with vacant housing; however, we believe this result helped clear potential threats of collinearity prior to the next step in our linear regression analysis.

Second, concerning linear correlation (Fig. 6), both the bus stop and access condition variables displayed correlations with the vacancy rate in each town. The proportion of houses within 200 m of a bus stop showed a positive correlation with the vacancy rate, whereas the proportion of houses with access conditions showed a negative correlation with the vacancy rate in each town.

Residences with larger floor areas were more likely to be vacant. Regarding living convenience, a greater distance to a bus stop and a lower proportion of houses with access conditions were correlated with a smaller vacancy rate. The bus stop variable exhibited a positive correlation with all residential size categories, with larger floor areas displaying a smaller ratio and reduced correlation. Conversely, the access condition variable demonstrated a negative correlation with all floor area categories, with larger floor areas showing a diminished ratio and correlation. Housing supply emerged as a robust explanatory factor for the proportion of vacant houses in each town in Toda City.

Finally, despite the variable S/F not displaying overt significance, its p-value of 0.067 was statistically meaningful, justifying its consideration as a factor influencing the vacancy rate.

**Table 5:** Correlation between house vacancy rate and neighbourhood characteristics including living convenience, housing supply, and policy support (*N* = 58).

Correlations											
Factors		Living convenience					Housing supply				Policy support
		300 F/N	S/F	P/F	C/F	B/N	A/N	15–64/N	65–99/N	100–150/N	RPD
VH/T	Pearson Cor.	-0.084	0.242	-0.054	-0.071	0.267 *	-0.317 *	0.076	0.448 **	0.283*	0.188
	Sig.	0.530	0.067	0.685	0.599	0.043	0.015	0.571	0.000	0.032	0.158

*p < 0.05, **p < 0.01.

#### Multiple regression analysis

We employed linear regression analysis to establish mathematical models, assessing the relationships and correlations between independent and dependent variables. We identified the positive or negative correlations between the 10 independent variables from three perspectives contributing to vacant houses, as shown in Table 6.

**Table 6:** Multiple regression analysis results (*N* = 58).

Analysis perspective	Factors	Beta	T	*P*	VIF	F	Significance	Adjusted *R* Square
Living convenience	300 F/N	0.061	0.419	0.677	1.676	3.228	0.003^b^	0.281
	S/F	0.419	3.089	0.003	1.459			
	P/F	-0.016	-0.130	0.897	1.167			
	C/F	-0.026	-0.204	0.839	1.272			
	B/N	0.162	1.174	0.246	1.507			
Housing supply	A/N	-0.360	-2.116	0.040	2.298			
	15–64/N	-0.083	-0.482	0.632	2.332			
	65–99/N	0.271	1.618	0.112	2.224			
	100–150/N	0.250	1.437	0.157	2.405			
Policy support	RPD	-0.189	-1.273	0.209	1.741			

The overall significance of the multiple regression model, as indicated by the statistical value F, was 3.228, with a corresponding p-value of 0.003, signifying the model’s significance. At 0.281, the adjusted R square indicated that the overall fit of the model was not good. This finding supports the earlier correlation analysis results showing that the relationships between the factors were relatively weak.

Overall, the model fit was relatively robust. In the S/F, the model showed a significantly positive influence, signifying that an increase in supermarket facilities correlates with a rise in the vacancy rate of each town in Toda City. Additionally, the coefficients of 65–99/N and 100–150/N on the vacancy rate were noteworthy, at 0.271 and 0.250, respectively. However, other factors (300 F/N, P/F, C/F, B/N, 15–64/N, RPD) lacked significance in the model, demonstrating weaker explanatory power. Analysis of the t- and p-values of each variable revealed that, among the 10 variables, S/F in housing convenience and A/N in housing supply had a high significance for the vacancy rate of each town in this research model.

The traditional multiple regression model failed to fully capture the spatial heterogeneity and dependency that influence housing vacancy rates in Toda City. This limitation may be due to the omission of geographic location and region-specific factors. Consequently, although variables such as living convenience and housing supply showed significance, their explanatory power may have been limited by the lack of spatial data features. Thus, we conducted an analysis from a spatial perspective and compared it with the statistical data analysis.

### Spatial distribution characteristics analysis

**Fig. 7:**
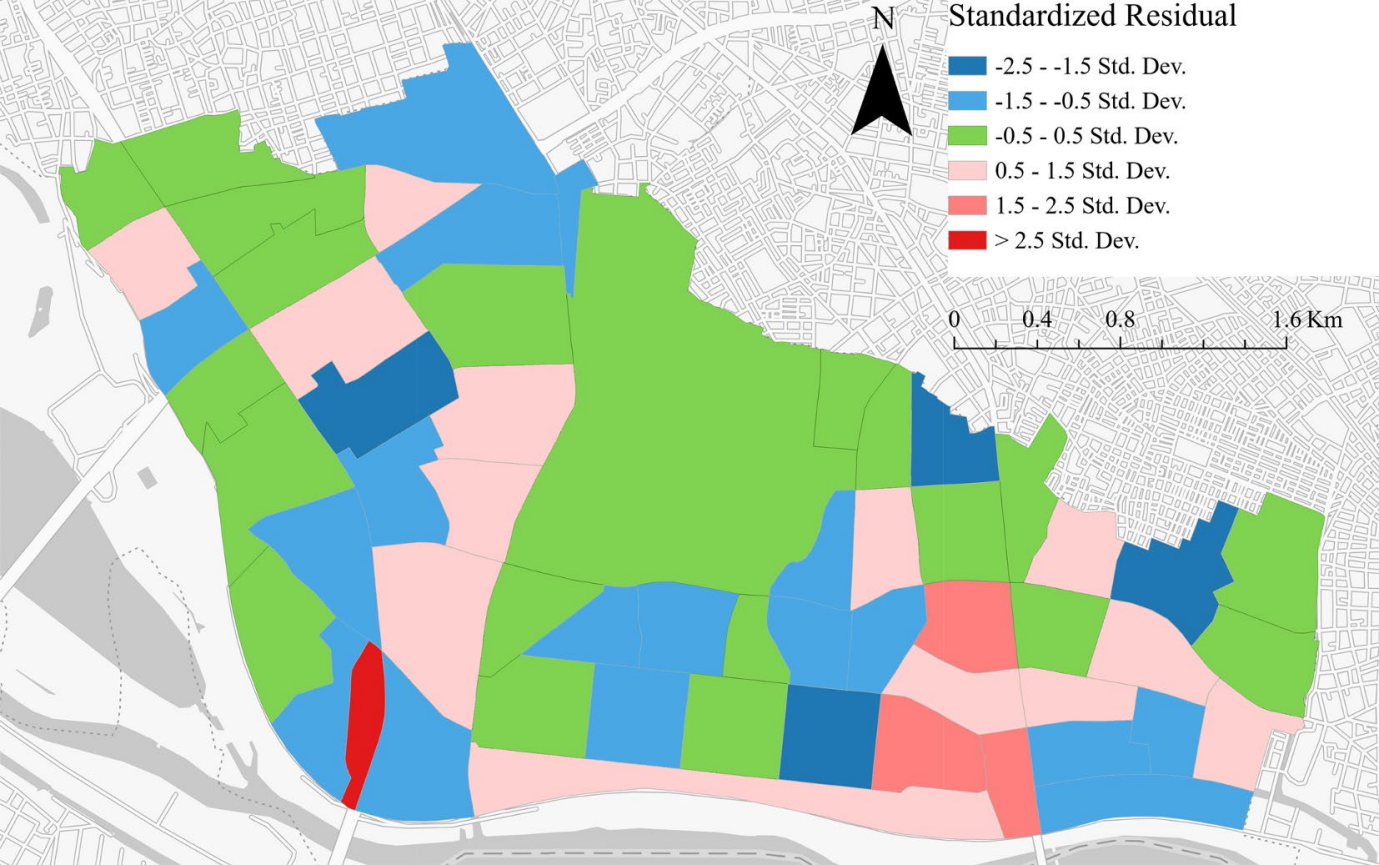
MGWR standardised residual distribution characteristics results.

This study analysed the spatial distribution characteristics using the MGWR approach. The results are presented in Table 7; Fig. 7. The MGWR analysis elucidated the factors influencing vacant housing within Toda City. The model’s statistical integrity was affirmed by an R-squared value of 0.7626, reflecting that a significant portion of the variance in the distribution of vacant housing was explained by the model. An adjusted R-squared of 0.6477, alongside an Akaike information criterion corrected (AICc) of 141.9366, attested to the model’s robust explanatory capacity. The sigma-squared value of 0.3494 and a sigma-squared maximum likelihood estimate (MLE) of 0.2374, with an effective degree of freedom of 39.4105, substantiated the model’s precision in depicting spatial variation.

**Table 7:** MGWR results and model diagnostics.

*R*-Squared	0.7626
Adjusted R-Squared	0.6477
AICc	141.9366
Sigma-Squared	0.3494
Sigma-Squared MLE	0.2374
Effective Degrees of Freedom	39.4105

In Fig. 7, blue represents areas where the model overpredicted the dependent variable, red indicates regions where the model underestimated the dependent variable, and green corresponded to areas where the model’s predictions closely matched the observed values. These observations align with the model’s goodness-of-fit, highlighting the degree to which the model accurately captured the spatial distribution of vacant houses in Toda City.

We also conducted separate analyses of the distribution characteristics and positive/negative correlations of each factor in the neighbourhoods of Toda City, aiming to compare them with the conclusions drawn from the data feature analysis. These analyses were performed to arrive at conclusions regarding the spatial distribution of significant factors in both data and space. The distribution of MGWR for each factor is shown in Table 8 and Appendix 2.

**Table 8:** Relationship between the number of significant MGWR towns and positive/negative correlations.

Analysis perspective	Influencing factor	Number of Significant Towns	Positive-Negative Correlation
Living convenience	300 F/N	4	+
	S/F	58	+
	P/F		
	C/F		
	B/N		
Housing supply	A/N	58	-
	15–64/N		
	65–99/N	5	+
	100–150/N	17	+
Policy support	RPD	1	-

Regarding the distribution characteristics of various factors in Toda City, the results revealed a positive correlation between living convenience variables, such as the number of facilities within a 300-meter radius (300 F/N) and the surface area of facilities (S/F), and vacant house distribution. This positive correlation was observed in 4 and 58 significant towns, respectively, suggesting that enhanced accessibility to amenities is inversely related to housing vacancy, likely due to increased attractiveness for occupancy.

Housing supply, denoted by A/N, inversely affected vacancy rates across 58 significant towns, suggesting that an excess in housing provision is a precursor to higher vacancies. Conversely, specific housing size categories, particularly 65–99/N and 100–150/N, were positively correlated with vacancies in 5 and 17 towns, respectively, indicating a likelihood for certain house sizes to remain unoccupied.

In terms of policy support, the RPD variable exhibited a negative coefficient in a single significant town, hinting at the potential efficacy of policy measures in fostering reduced vacancy rates. This suggests that targeted policy support, particularly in designated residential promotion districts, may be instrumental in mitigating vacancy levels.

There were commonalities between the statistical data analysis and spatial analysis. The statistical analysis identified the most closely related factors affecting the distribution of vacant houses in Toda City. We focused on further analysis of the S/F (living convenience) and A/N (housing supply) factors to understand their forms and influence in Toda City.

### Scatterplot analysis of high-significance factors and vacant houses

**Fig. 8:**
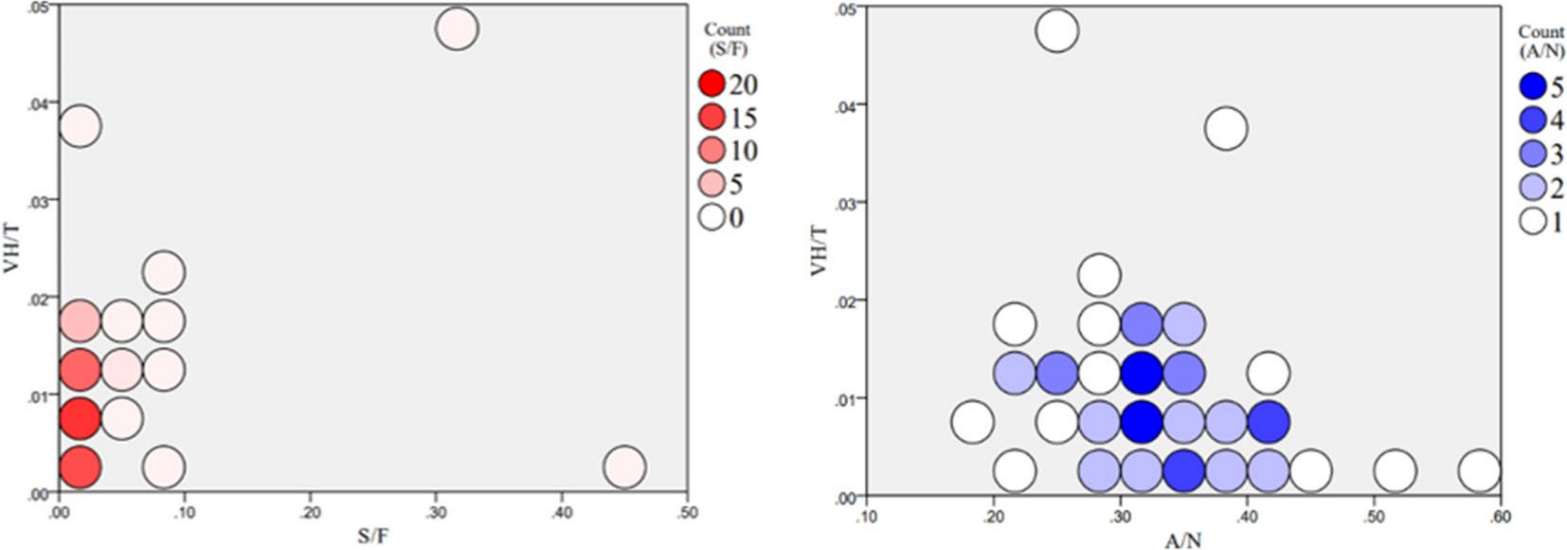
Scatterplot and centroid coordinate analysis of S/F and A/N.

Combining the linear correlation analysis with the results of the multiple linear regression analysis, we aimed to understand the relationship between the distribution of vacant houses in Toda City and the two most relevant factors: S/F (living convenience) and A/N (housing supply).The investigation involved the extraction of these two factors individually, followed by a scatter state analysis correlating each town’s vacant house rate, as represented in Fig. 8.

Regarding S/F, the ratio of supermarket area to residential area in each district of Toda City predominantly fell within the interval of low housing vacancy rates (0–10%), with a concentrated trend of the size of the ratio’s data points mainly within the 0–15% range. This suggests that in areas with a lower vacancy rate in Toda City, the distribution of the ratio of supermarket area to residential area is concentrated and more correlated with the vacancy rate when the ratio falls within the range of 0 to 0.15.

Concerning A/N, the scatter plot primarily spread across the higher vacancy rate interval (30–40%), with the proportion of residences with road access distributed in the 5–15% range for each town. This indicates that in areas of Toda City with a higher vacancy rate, the proportion of residences with road adjacency is more correlated with the vacancy rate when it falls within the 5–15% range. The results imply that when the vacancy rate in a town in Toda City is relatively low, it may be more correlated with the distribution of convenient supermarket facilities. Conversely, when the vacancy rate is higher, it may be more correlated with the proportion of residences within that town having road adjacency.

## Discussion

This study focused on traditional data analysis methods, such as correlation analysis, multiple regression analysis, and cluster analysis, and compared these with spatial data analysis to evaluate outcomes. Our findings revealed that the MGWR model (adjusted R-Squared: 0.6477) outperformed the multiple regression model (adjusted R-Squared: 0.281), highlighting the significance of spatial distribution characteristics over traditional statistical data modeling for Toda City. This underscores the importance of spatial factors when analyzing vacancy rates, especially in satellite cities.

The analysis identified the area ratio of supermarkets to houses (S/F) and road adjacency conditions (A/N) as key determinants impacting vacancy rates. These factors consistently appeared significant in both analytical methods, with S/F showing a positive correlation and A/N showing a negative correlation with vacancy rates. This finding contrasts with previous research by Baba and Hino (2019)^20^ and Baba and Asami (2017)^19^, who reported different results regarding adjacency conditions. The innovative approach of our study, focusing on the ratio of supermarket facility area to residential area, offers a fresh perspective, aligning with some studies on urban convenience in Japan and Taiwan (Baba & Asami, 2017; Chiang et al., 2015)^19,21^ but differing from evidence in Tokyo (Gu et al., 2016)^1^. Additionally, the negative correlation between road distance from houses and vacancy suggests an insufficient consideration of surrounding facilities in Toda City, potentially influenced by the advanced state of urban construction roads.

The results underscore the critical role of urban convenience and accessibility in shaping vacancy rates, suggesting that policies aimed at enhancing these factors could reduce vacancies. The findings have practical implications for urban planners and policymakers, emphasizing the need for strategies that integrate spatial characteristics with traditional urban planning metrics to address vacancy issues effectively.

However, the study has several limitations. The primary limitation is its narrow geographical scope, focusing solely on Toda City, one of the satellite cities of Tokyo metropolis. This limited sample size may not represent other similar satellite cities, reducing the generalizability of the findings. Although the examined factors of living convenience, housing supply, and policy support encompassed 10 influencing variables, they may not comprehensively represent these domains. While the publicly available data employed is recent and comprises well-organized mapping data, future studies should integrate and refine data from diverse sources for more precise conclusions. Furthermore, the selected factors did not sufficiently explain the variance in vacancy rates within the model, indicating that broader socio-economic influences, including land use, land price, transportation convenience, and overall quality of life, may be at play, extending beyond the 10 variables analyzed.

Nevertheless, this study is significant because discussions on urban vacancy rates in countries other than Japan, such as China, have primarily focused on resident numbers, income, and the geographical orientation of urban locations (Yang, Xiao et al., 2023; Zhang et al., 2016; Dong et al., 2021)^33,76,77^. With the emergence and decentralization of global cities over the past few decades, future research should explore the impact of land use changes on vacant houses, as highlighted by Zhang et al. (2023)^17^ and Li et al. (2019)^78^. The significance of satellite cities surrounding major metropolitan areas becomes more pronounced during ongoing phases of urban development, not only in Japan but also in countries in the Global South, including Egypt (Salem et al., 2020)^79^, Ethiopia (Koroso et al., 2021)^80^, and Iran (Sabet Sarvestani et al., 2011)^81^. Well-developed satellite cities within comprehensive metropolitan regions exhibit closer interactions with the city center (Yang, Wang et al., 2023)^82^. As Kidokoro et al. (2023)^50^ posited, the emergence of ‘urban suburbia’ might also be a possible factor leading to the urban decline and housing vacancy in surrounding satellite cities. A phenomenon of multipolarity in satellite cities may even emerge to stimulate local vitality (Chen et al., 2019)^83^. Considering the internal dynamics of cities, it is also imperative to consider a broader range of urban facilities, as suggested by Campos et al. (2018)^84^ and Abe et al. (2014)^85^. Gentrification, driven by housing demand and supply, is also an important aspect (Lee & Newman, 2021)^63^.

In this study, we also visually illustrated the geographic distribution of the MGWR model’s residuals and the localized significance of each neighborhood characteristic, thereby augmenting the interpretative value of the statistical findings. Future research should explore the internal mechanism of vacancy rates from the perspective of population mobility, economic changes, and the impact of urban planning decisions, considering the complex nature of satellite cities. Finally, compared to other similar studies (Deng et al., 2023; Pan et al., 2020)^54,86^, the explanatory power of vacant houses in this study remained relatively limited. It is crucial to acknowledge that vacant houses may be subject to broader socio-economic influences.

## Conclusion

This study sought to examine urban vacancy dynamics in Toda City, a satellite city of the Tokyo metropolis. By analysing the correlation between vacant house distribution and factors such as living convenience, housing supply, and policy support, our findings provide valuable insights for urban planning and development in satellite cities. The results offer a strategic foundation for mitigating housing vacancies, fostering regional vitality, and improving the living environment. The study found a significant positive correlation between vacant house distribution and the proportion of supermarket area, while a significant negative correlation was observed with the proportion of buildings that feature accessibility enhancements. This suggests that areas with lower vacancy rates have a stronger relationship between the ratio of supermarket area to residential space. On the other hand, in high-vacancy areas, the proportion of buildings connected to roads was more prominent. 

This study underscores the significance of spatial factors in understanding urban vacancy dynamics, especially in satellite cities where development patterns can vary greatly from those in urban centres. Through MGWR analysis, the research demonstrates how spatial data can offer a nuanced understanding of vacancy trends, providing valuable insights for urban planners and policymakers. By highlighting the importance of living convenience and accessibility, the study suggests that improving these factors can effectively reduce vacancy rates and enhance urban living conditions. The findings lay an important foundation for future strategies aimed at revitalising satellite cities, ensuring they positively contribute to the broader metropolitan region.

Overall, this study reveals there exists a complex relationship between urban planning, accessibility, and vacancy rates, paving the way for proactive strategies and effective vacant property management. Future studies can build on these results by exploring additional variables and applying the insights across various urban contexts.

## Electronic supplementary material

Below is the link to the electronic supplementary material.

Supplementary Material 1

## Data Availability

The datasets used and analysed during the current study available from the corresponding author on reasonable request.
