# Supplementary figures and images for: Effects of neighbourhood characteristics on the distribution of vacant houses in Toda city, a satellite city of Tokyo metropolis of japan
Source: Sci Rep. 2024 Oct 28;14:25794. doi: 10.1038/s41598-024-74663-3 (PMC11519946; doi:10.1038/s41598-024-74663-3)

## Slide 1
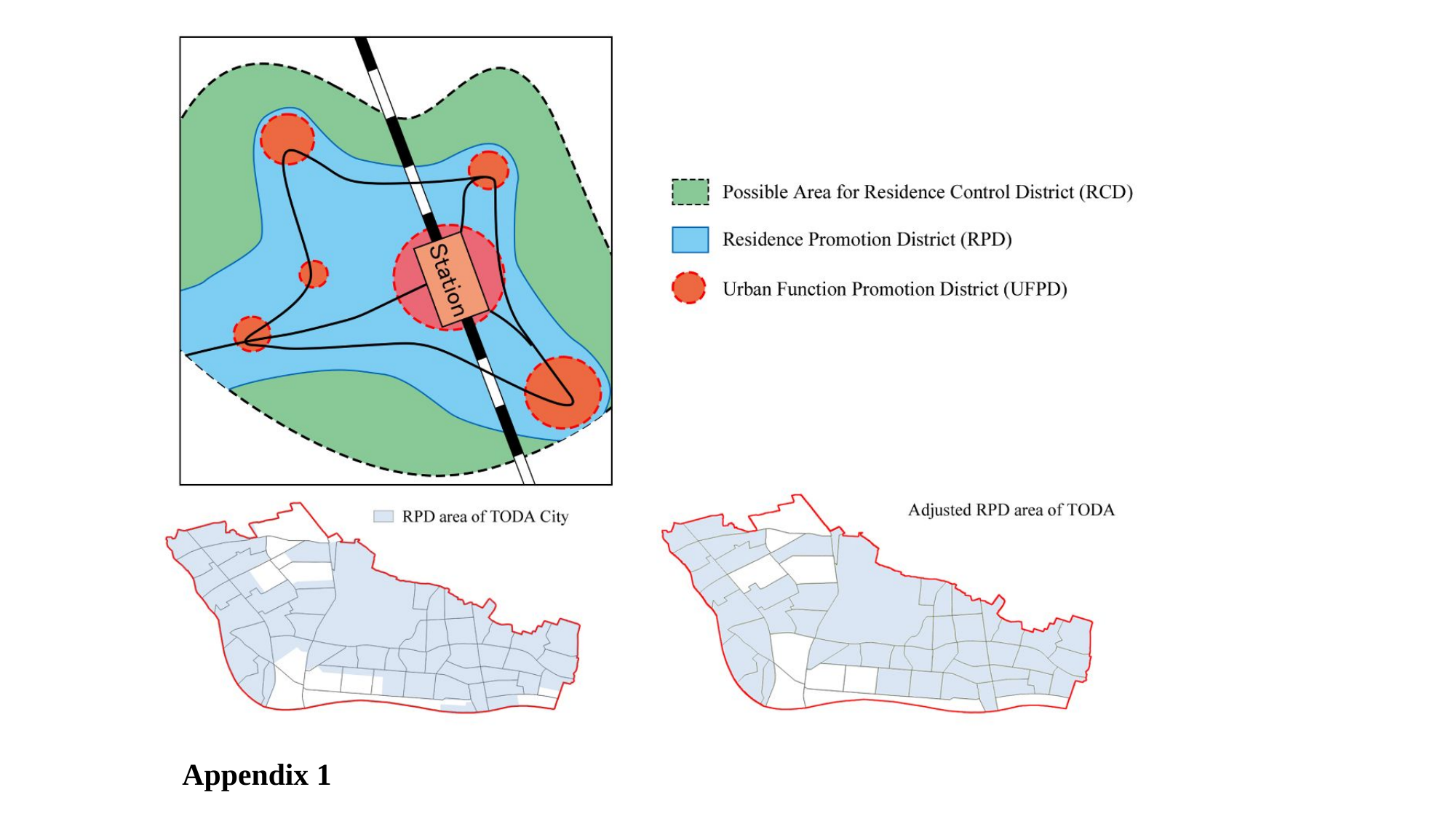

Appendix 1

## Slide 2
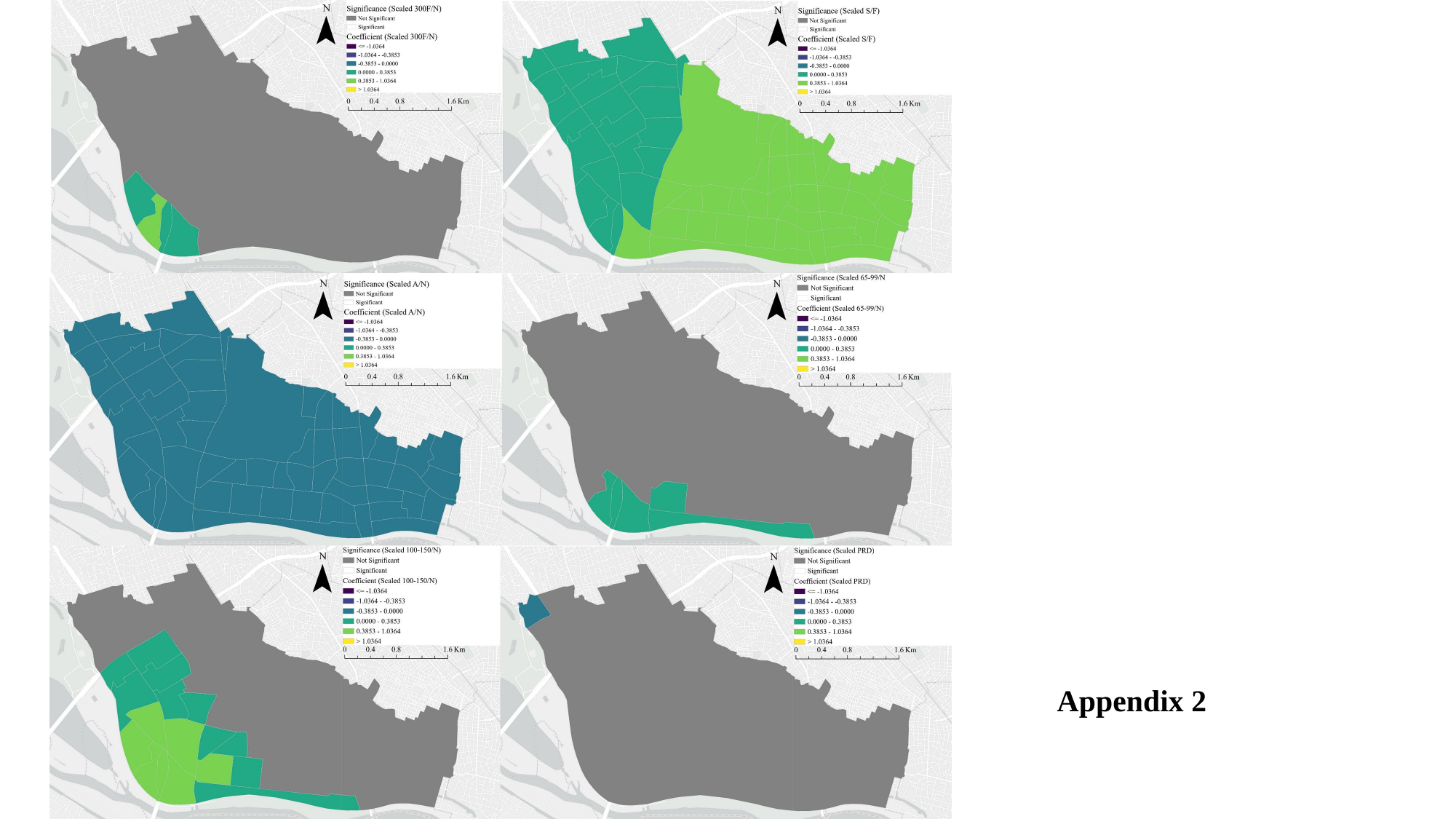

Appendix 2

## Slide 3
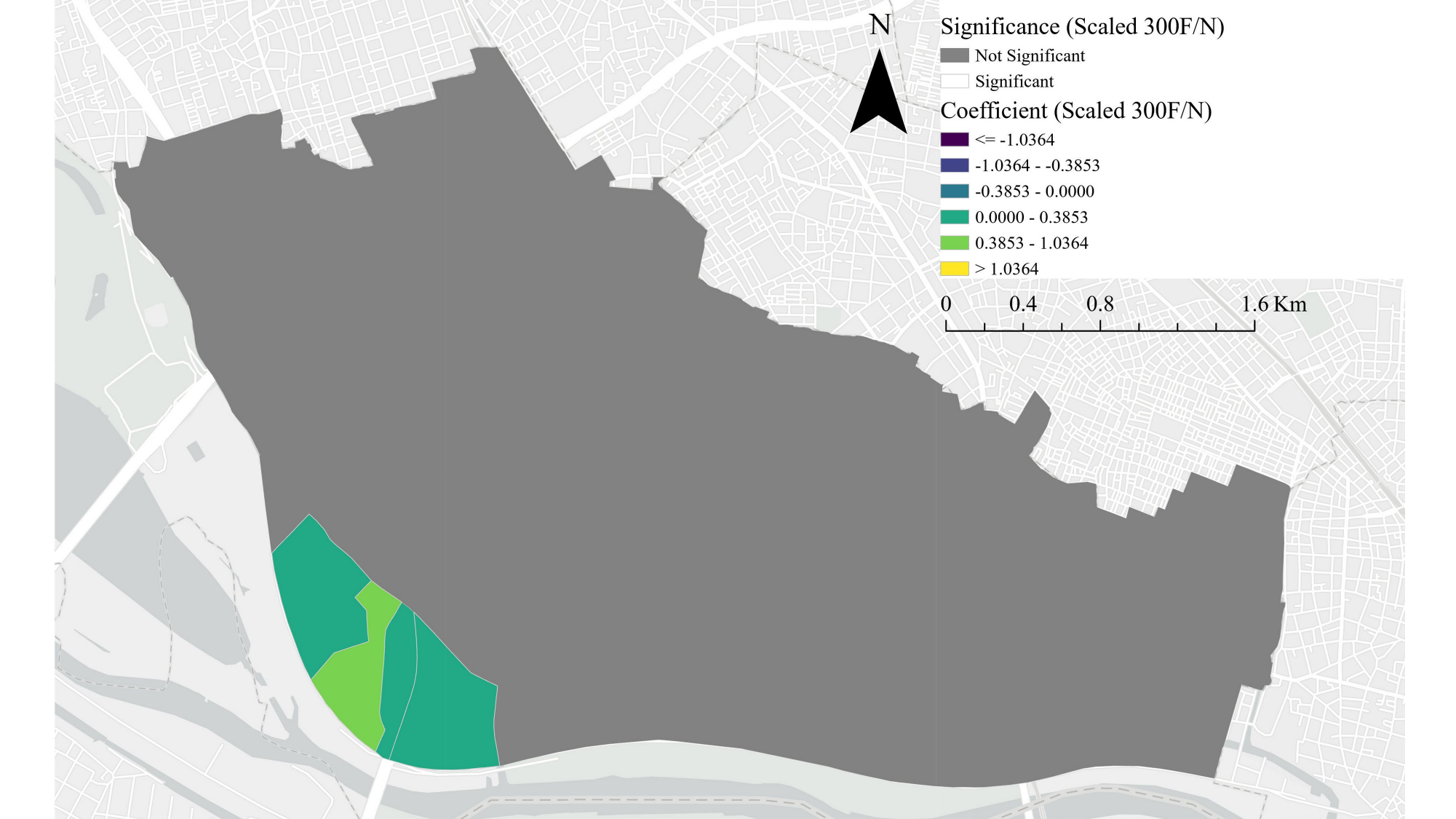

## Slide 4
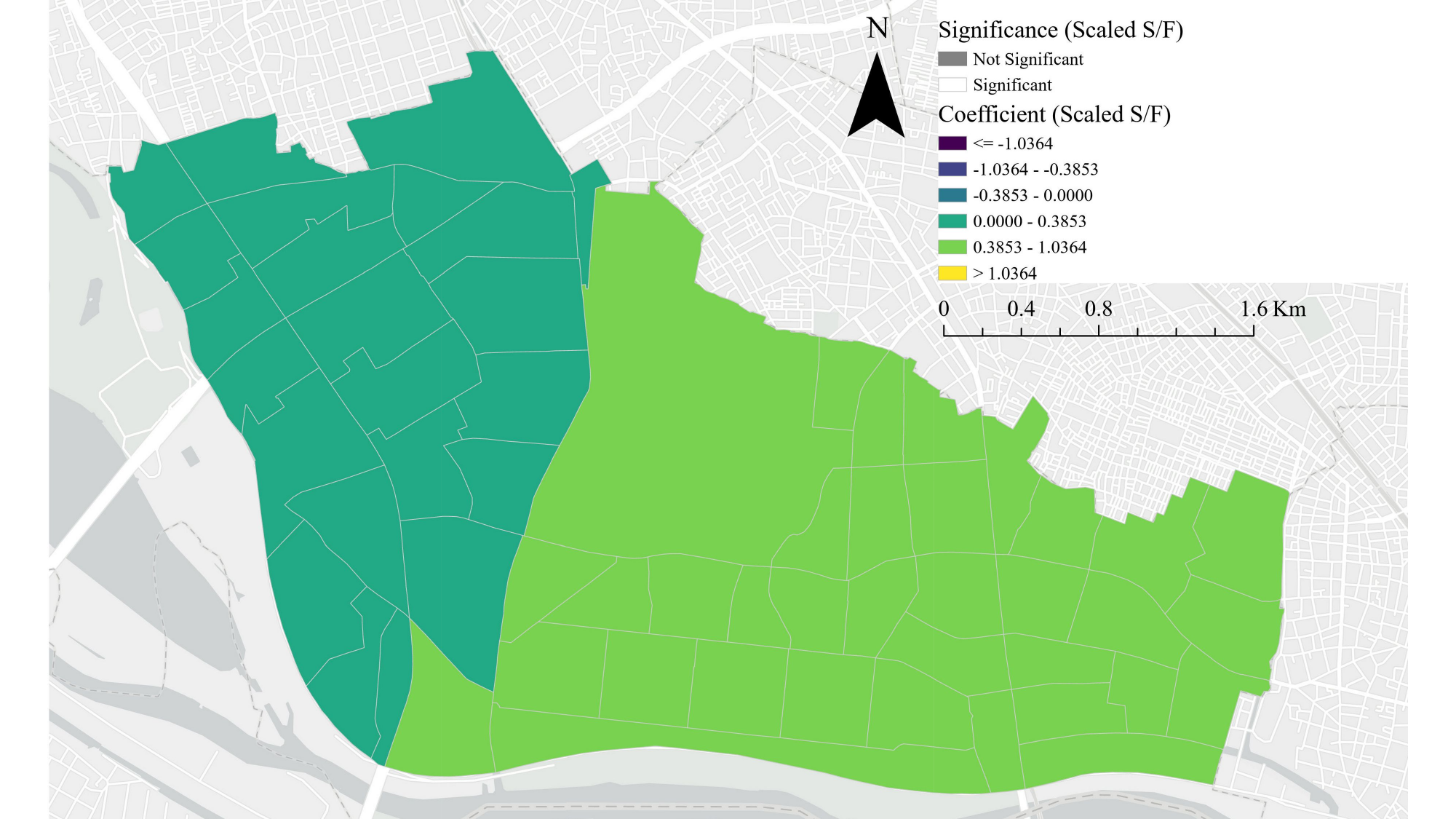

## Slide 5
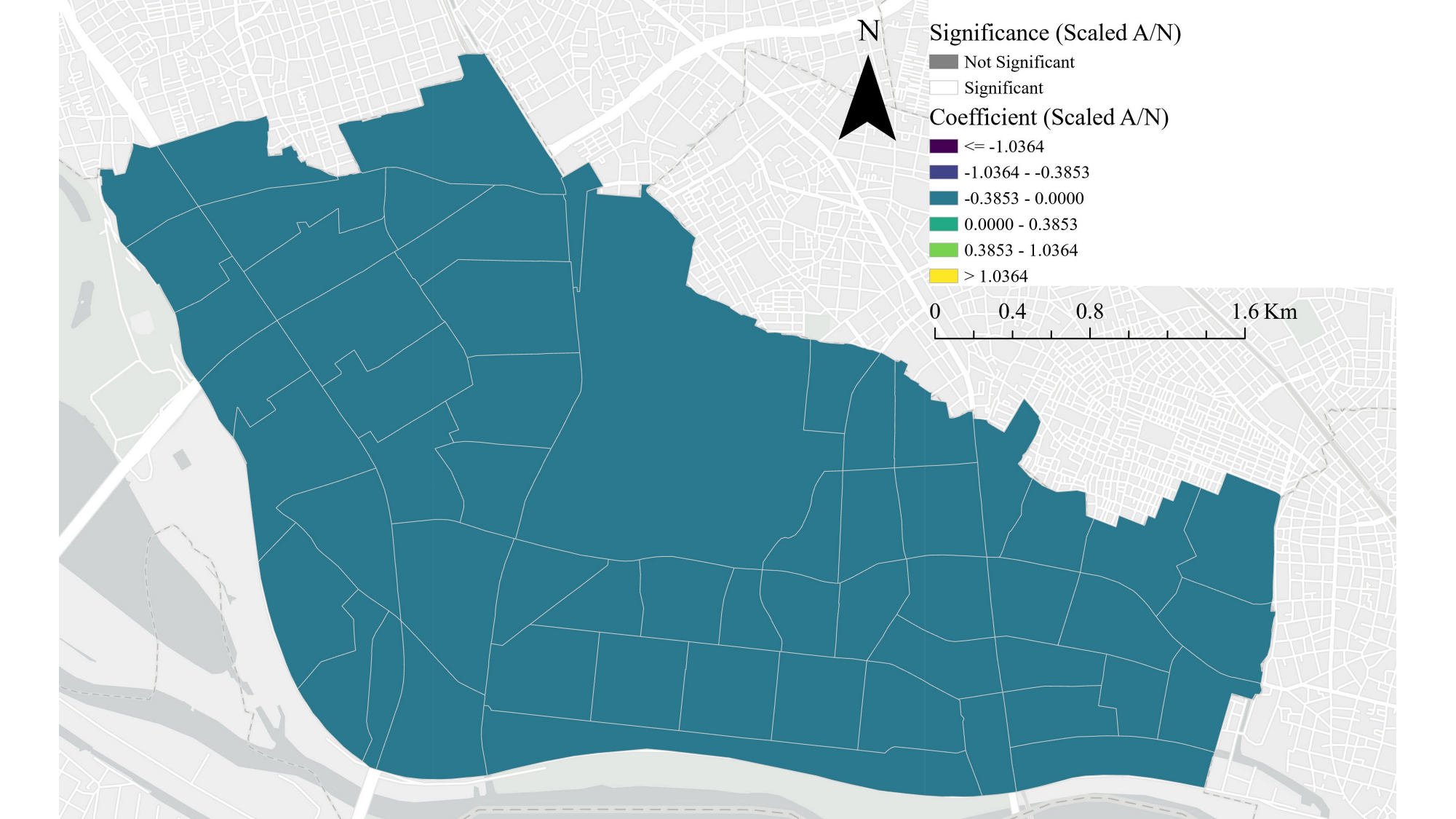

## Slide 6
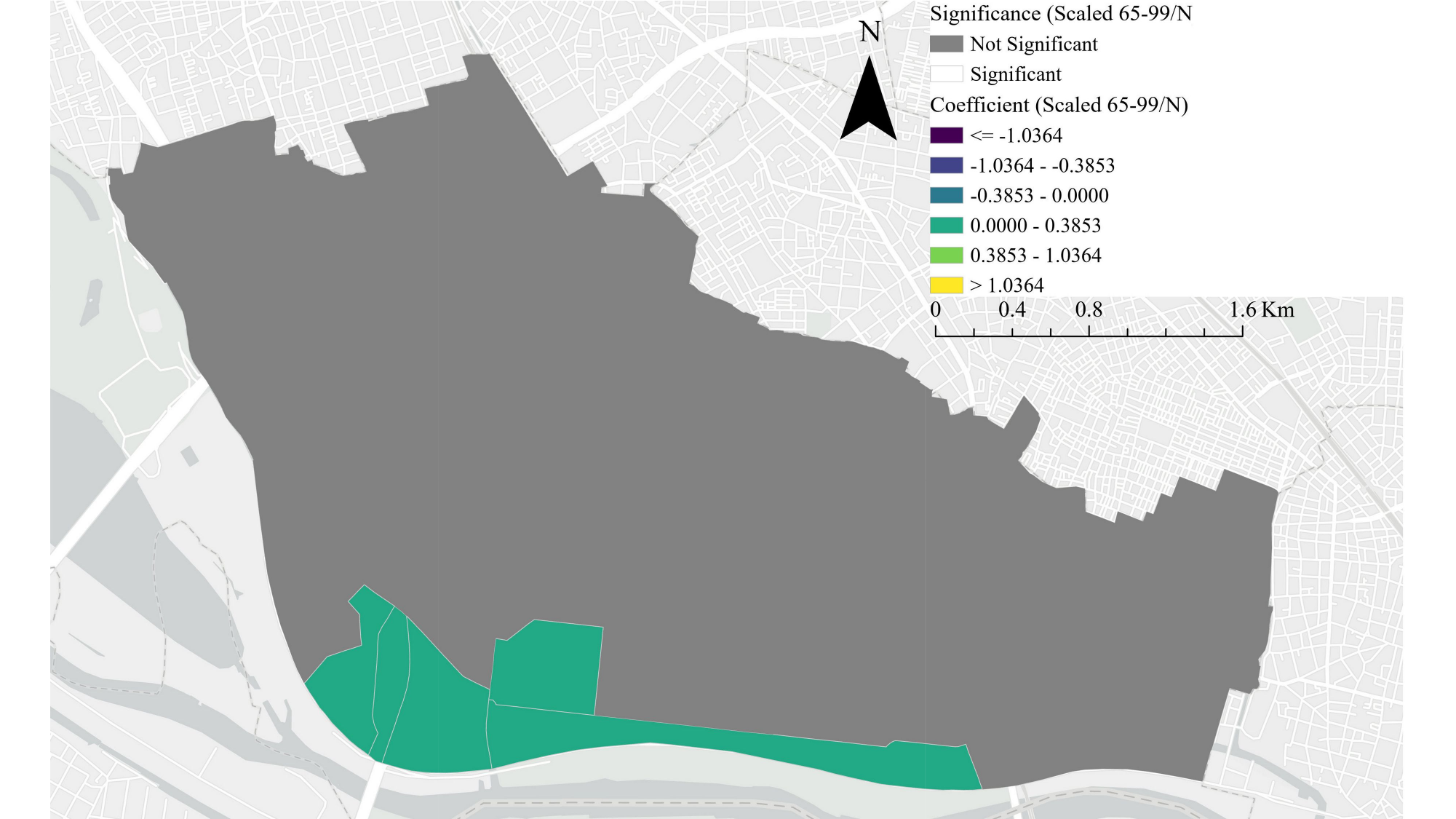

## Slide 7
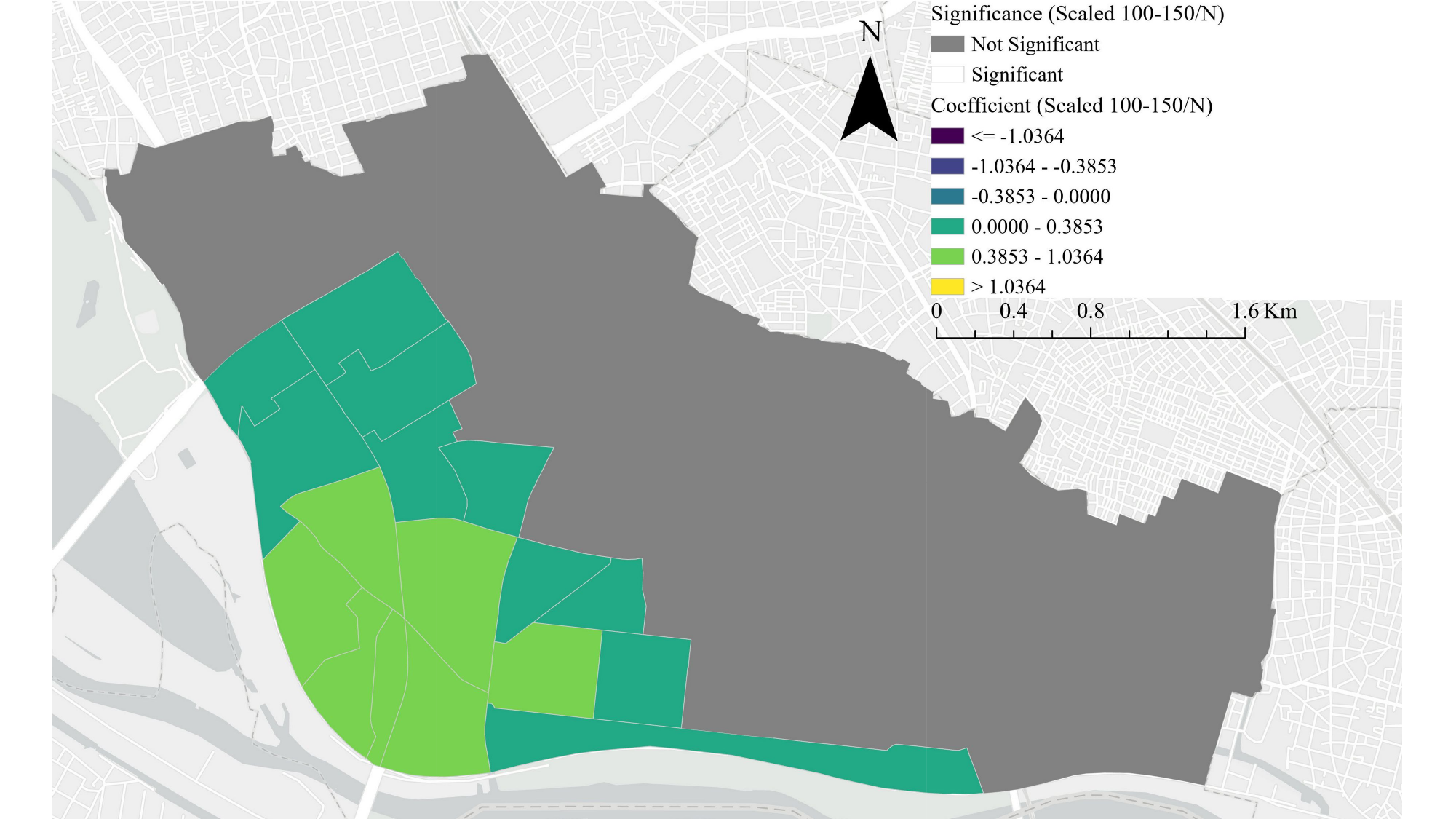

Supplement: Supplementary file 1 — Supplementary Material 1 [file 41598_2024_74663_MOESM1_ESM.pptx]
